# The Effect of Parental Awareness on Adolescent Stress Management

**DOI:** 10.7759/cureus.108522

**Published:** 2026-05-08

**Authors:** Chetna Sharma, Poornima Shah

**Affiliations:** 1 Department of Home Science, Government Kamla Raja Girls Post Graduate Autonomous College, Gwalior, IND

**Keywords:** adolescent stress, family dynamics, parental awareness, resilience, stress management

## Abstract

Introduction and aim: Adolescence is a vulnerable period marked by significant stress, with parental awareness potentially playing a crucial role in shaping adolescents’ ability to manage stress. This study aimed to assess the effect of parental awareness on adolescent stress and stress management.

Material and methods: A cross-sectional analytical study was conducted among 400 adolescents aged 11-18 years selected from schools in Gwalior district, India, from April 2022 to January 2024. Data were collected using the Adolescent Stress Scale (ADOSS) to assess stress, a self-constructed questionnaire for parental awareness, and the Brief Resilience Scale (BRS-6) to evaluate stress management (resilience). Descriptive and inferential statistics, including independent t-tests, one-way analysis of variance (ANOVA), Pearson correlation, and multivariate regression, were applied.

Results: Mean stress score was 43.4 ± 10.1, parental awareness score was 65.1 ± 7.7, and stress management score was 74.2 ± 11.4. Significant differences in stress levels were observed across gender, school type, family type, parental education, and socioeconomic status (p<0.05). Pearson correlation analysis revealed a significant, moderate negative relationship between parental awareness and adolescent stress, indicating that higher parental awareness is associated with lower adolescent stress. Higher parental awareness was significantly associated with lower stress (β = -0.35, p = 0.001) and better stress management (β = 0.32, p = 0.001). Multivariate regression models explained 32% and 38% of variance in stress and stress management, respectively. Female sex, nuclear family structure, and lower socioeconomic status emerged as significant risk factors for higher stress and poorer coping.

Conclusion: Parental awareness significantly influences adolescent stress and stress management. Family-centered interventions that enhance parental awareness may serve as an effective strategy to mitigate adolescent stress and promote resilience.

## Introduction

Adolescence is a critical developmental phase characterized by rapid physical, emotional, and social changes [1]. Historically, the concept of adolescence as a distinct stage of life was first systematically described by G. Stanley Hall in the early 20th century, who referred to it as a period of “storm and stress.” Since then, research in developmental psychology and public health has consistently highlighted adolescence as a vulnerable period for the onset of psychological stress and related disorders [2]. In recent decades, increasing academic pressure, social expectations, digital exposure, and changing family dynamics have further intensified stress levels among adolescents worldwide [3].

Adolescent stress is defined as the psychological and physiological response to perceived challenges or demands that exceed an individual’s coping resources. Studies indicate that stress during adolescence can arise from multiple domains, including academic performance, peer relationships, body image concerns, and family environment [4]. Chronic stress during this stage has been associated with adverse outcomes such as anxiety, depression, substance abuse, and impaired academic performance [5]. Neurobiological research suggests that the developing brain, particularly the prefrontal cortex and limbic system, is highly sensitive to stress, making adolescents more susceptible to emotional dysregulation [6].

Effective stress management is therefore crucial in mitigating these negative outcomes. Stress management strategies among adolescents include cognitive-behavioral techniques, mindfulness practices, physical activity, and social support systems [7]. School-based interventions and life skills education programs have demonstrated positive effects in enhancing coping mechanisms. However, the effectiveness of these strategies often depends on the broader psychosocial environment, particularly the role of family [8].

Parental awareness plays a pivotal role in shaping adolescents’ ability to manage stress. Parents who are knowledgeable about the sources, symptoms, and consequences of stress are better equipped to provide emotional support, guidance, and appropriate interventions [9]. Research has shown that positive parenting practices, such as open communication, empathy, and supportive monitoring, are significantly associated with lower stress levels and better coping outcomes in adolescents. Conversely, lack of parental awareness or involvement may exacerbate stress and hinder effective stress management [10].

Despite the growing body of literature on adolescent stress and coping strategies, there remains a notable gap in understanding the direct relationship between parental awareness and adolescent stress management. Many studies have examined these variables independently, but limited research has explored their combined and interactive effects, particularly across diverse sociocultural contexts. Furthermore, sex-based differences in stress perception and coping, as well as the mediating role of stress management techniques, require further empirical investigation. Therefore, the present study aimed to address these gaps by examining the effect of parental awareness on adolescent stress and stress management, thereby contributing to a more comprehensive understanding of this critical public health issue.

## Materials and methods

Study design

The present study was designed as a cross-sectional analytical study aimed at assessing the effect of parental awareness on adolescent stress and stress management. A questionnaire-based approach was employed to collect quantitative data from adolescents and their parents. Relationships between variables such as parental awareness, stress levels, and stress management were analyzed at a single point in time, allowing for comparison across sex and age groups.

Study setting

This study was conducted at Government Kamla Raja Girls Post Graduate Autonomous College, Gwalior, India, from April 2022 to January 2024 and included schools located in three sub-districts of Gwalior district, namely Gwalior, Murar, and Lashkar. Both government and private higher secondary and senior secondary schools affiliated with the Madhya Pradesh Board and the Central Board of Secondary Education were included. The study was approved by the Institutional Ethical Committee (approval number: KRGC/IEC/2022/04/028) and followed the guidelines of the Declaration of Helsinki 2017.

Inclusion and exclusion criteria

Adolescents aged between 11 and 18 years were considered for inclusion and categorized into the following three groups: early adolescence (11-12 years), middle adolescence (13-16 years), and late adolescence (17-18 years). Students enrolled in selected schools and present on the day of data collection were included. Those who provided assent and had parental consent were considered eligible. Students with known psychological disorders, those undergoing psychiatric treatment, or those unwilling to participate were excluded. Incomplete questionnaire responses were also excluded from the final analysis to maintain data integrity and validity.

Sample size estimation

A total sample size of 400 adolescents was included in this study. The sample size was determined to ensure 80% statistical power and a 5% alpha error for detecting the association between parental awareness and adolescent stress (r = 0.34) [11]. Schools were selected using stratified sampling from government and private institutions across three sub-districts. Within selected schools, students were chosen using systematic random sampling (every fifth student from the school register). This approach ensured representation across gender, school type, and socioeconomic backgrounds (by the modified Kuppuswamy Socioeconomic Status (SES) 2025 scale, a license-free tool) [12].

Procedure for stress calculation

The Adolescent Stress Scale (ADOSS) is novel and subjective [13]. It is short (with 20 questions) and includes stressors that Indian adolescents encounter daily. Adolescent stress levels were assessed using the ADOSS. The scale consists of standardized items measuring psychological stress experienced by students. The ADOSS asks respondents about their thoughts and feelings over the last two weeks. Each item is rated on a four-point scale ranging from 0 for “never” to 3 for “nearly every day.” ADOSS scores are calculated by summing the scores obtained on all scale items except two positively worded items (items 3 and 12) that need reverse scoring (e.g., 3 = 0, 2 = 1, 1 = 2, and 0 = 3). Thus, the total score is 60, with higher scores indicating high stress. The cumulative score indicated the level of stress, which was further categorized into low (0-20), medium (21-40), and high stress (41-60) levels for analysis.

Procedure for parental awareness calculation

Parental awareness was assessed using a self-constructed questionnaire consisting of 25 items. Each item had the following three response options: never (1), sometimes (2), and always (3). The total score ranged from 25 to 75. Based on score distribution, parental awareness was categorized into low (25-34), medium (35-63), and high (64-75) levels. Parental awareness was assessed using a self-constructed questionnaire consisting of 25 items (table in appendix).

Procedure for stress management (resilience) score calculation

Stress management was assessed using resilience (six-item Brief Resilience Scale {BRS-6}) as a proxy indicator. Resilience was evaluated using the six-item Brief Resilience Scale (BRS), a widely validated self-report instrument suitable for adolescent populations [14]. The scale assesses an individual’s ability to cope with stress and adversity. Each item is rated on a 0-100-point visual analog scale (VAS), yielding a total possible score between 0 and 100. Higher total scores reflect greater psychological resilience and a stronger capacity to manage stress effectively. The score indicated the level of resilience, which was further categorized into low (0-30), medium (31-60), and high (61-100) stress levels for analysis. BRS-6 is freely available with no license fee.

Quality assessment of the questionnaire

ADOSS and BRS-6 are pre-validated questionnaires with Cronbach's alpha values of 0.85-0.89. The self-constructed questionnaires for parental awareness underwent content validation by subject experts in psychology and education. A pilot study was conducted to test clarity, reliability, and feasibility. Internal consistency was assessed using Cronbach’s alpha, ensuring 0.87 reliability. Necessary modifications were made based on feedback. Standardized administration procedures were followed to minimize bias and ensure uniform data collection across participants.

Outcome variables

The primary outcome variables included adolescent stress levels and stress management ability. Stress levels were measured using a standardized scale, while stress management was assessed through a structured questionnaire. The main independent variable was parental awareness, categorized into three levels. Additional variables included sex, age group, type of school, and socioeconomic and educational status of parents. These variables were analyzed to determine their influence on stress and coping mechanisms among adolescents.

Statistical analysis

Data were entered and analyzed using IBM SPSS Statistics version 25.0 (Armonk, NY: IBM Corp.). Descriptive statistics, including mean, standard deviation, frequency, and percentage, were used to summarize the data. Data were assessed and confirmed for normal distribution with the Shapiro-Wilk test. Inferential statistics, such as the independent t-test, were applied to compare stress levels across gender. One-way analysis of variance (ANOVA) was used to assess differences among multiple groups. The association between parental awareness, stress, and stress management was evaluated using correlation analysis (Pearson’s correlation coefficient). Multiple linear regression analysis was performed to determine the predictive effect of parental awareness on stress and stress management. Multicollinearity was checked using the variance inflation factor (VIF) (<5). A p<0.05 was considered statistically significant.

## Results

The study enrolled 400 adolescents, predominantly male adolescents from private schools and nuclear families. The age distribution showed middle adolescence as the largest group, with 199 (49.8%) participants, followed by the early and late stages. Parental education was secondary in nearly half, with higher and primary levels balanced. A total of 188 (47%) participants were from low- and middle-socioeconomic status groups, and 212 (53%) were from upper- and high-class groups (Table 1).

**Table 1:** Sociodemographic characteristics of study population. Data are presented as frequency (n) and percentage (%).

Characteristics	Category	n (%)
Age group (years)	Early (11-12)	124 (31.0)
	Middle (13-16)	199 (49.8)
	Late (17-18)	77 (19.2)
Sex	Adolescent boys	232 (58.0)
	Adolescent girls	168 (42.0)
School type	Government school	65 (16.2)
	Private school	335 (83.8)
Family type	Joint family	72 (18.0)
	Nuclear family	328 (82.0)
Parental education	Primary	102 (25.5)
	Secondary	193 (48.2)
	Higher	105 (26.3)
Socioeconomic status	Low/lower middle	188 (47.0)
	Upper middle/high	212 (53.0)

Descriptive statistics for outcome variables showed moderate mean stress levels (ADOSS: 43.4 ± 10.1, range 8-58, Cronbach's α = 0.89), fair parental awareness (65.1 ± 7.7, range: 32-75, α = 0.87), and good stress management (BRS-6: 74.2 ± 11.4, range: 25-98, α = 0.85). All scales demonstrated strong internal reliability (α > 0.85). These findings indicate acceptable psychometric properties in this adolescent cohort, with room for stress reduction interventions to enhance resilience and potentially mitigate associated anxiety-related habits. The Pearson correlation analysis revealed a significant moderate negative relationship between parental awareness and adolescent stress, indicating that higher awareness is associated with lower stress levels. Additionally, parental awareness showed a significant positive correlation with stress management, suggesting improved coping abilities. Stress management was negatively correlated with stress scores, highlighting its protective role. Overall, increased parental awareness contributes to better stress management and reduced stress among adolescents (Table 2).

**Table 2:** Pearson correlation analysis between outcome variables. *P<0.05 was considered statistically significant. Data are presented as mean ± standard deviation (SD). Pearson correlation coefficient (r) was used to assess the strength and direction of associations between variables. The symbol “-” indicates non-applicable values or self-correlation within the same variable, where statistical comparison is not required. ADOSS: Adolescent Stress Scale; BRS-6: Brief Resilience Scale

Pearson correlation	Mean ± SD	Stress score (ADOSS)		Parental awareness score		Stress management score (BRS-6)	
		r-Value	p-Value	r-Value	p-Value	r-Value	p-Value
Stress score (ADOSS)	43.4 ± 10.1	1	-	-0.46	<0.001*	-0.52	<0.001*
Parental awareness score	65.1 ± 7.7	-0.46	<0.001*	1	-	0.58	<0.001*
Stress management score (BRS-6)	74.2 ± 11.4	-0.52	<0.001*	0.58	<0.001*	1	-

Stress scores (ADOSS) were highest in government school (47.1 ± 8.5) and parental primary education groups (45.2 ± 10.6), lowest in higher education (41.5 ± 9.7) and upper socioeconomic status (41.9 ± 9.7) groups. Parental awareness peaked in higher education (67.8 ± 6.8) and private schools (66.1 ± 7.2), was lowest in government schools (62.3 ± 8.2), and was low in lower-middle socioeconomic status (63.8 ± 7.9). Stress management was superior in private schools (76.0 ± 11.0), higher education (76.8 ± 10.5), and boys (75.5 ± 11.0) versus girls (72.3 ± 11.7). Girls and lower socioeconomic status/nuclear family adolescents showed elevated stress with poorer coping (Table 3). These gradients highlight socioeconomic and educational disparities driving stress vulnerability, potentially amplifying mental health challenges among disadvantaged subgroups, and underscore the need for school-based interventions.

**Table 3:** Outcome scores by key variables. Data are presented as mean and standard deviation (SD). ADOSS: Adolescent Stress Scale; BRS-6: Brief Resilience Scale

Characteristics	Category	Stress score (ADOSS) (mean ± SD)	Parental awareness score (mean ± SD)	Stress management score (BRS-6) (mean ± SD)
Age group (years)	Early (11‑12)	42.5 ± 9.8	65.2 ± 7.4	72.5 ± 11.8
	Middle (13‑16)	44.1 ± 10.2	64.8 ± 8.1	74.8 ± 11.0
	Late (17‑18)	43.2 ± 9.5	65.5 ± 7.0	76.2 ± 11.3
Sex	Adolescent boys	42.5 ± 9.8	65.0 ± 7.6	75.5 ± 11.0
	Adolescent girls	45.0 ± 10.4	64.5 ± 7.9	72.3 ± 11.7
School type	Government school	47.1 ± 8.5	62.3 ± 8.2	70.5 ± 10.5
	Private school	42.3 ± 9.7	66.1 ± 7.2	76.0 ± 11.0
Family type	Nuclear family	45.8 ± 10.5	64.2 ± 8.0	73.0 ± 11.8
	Joint family	42.9 ± 9.9	65.3 ± 7.5	74.8 ± 11.4
Parental education	Primary	45.2 ± 10.6	62.5 ± 8.2	72.0 ± 12.2
	Secondary	43.1 ± 9.9	64.8 ± 7.5	74.2 ± 11.2
	Higher	41.5 ± 9.7	67.8 ± 6.8	76.8 ± 10.5
Socioeconomic status	Low/lower middle	45.0 ± 10.3	63.8 ± 7.9	73.5 ± 11.8
	Upper middle/high	41.9 ± 9.7	66.4 ± 7.4	75.0 ± 11.0

Comparative analysis showed that significant differences emerged across demographics (Table 4). Stress scores varied by sex (p = 0.015; boys lower), school type (p = 0.001; government higher), family type (p = 0.035; nuclear higher), parental education (p = 0.027; primary highest), and socioeconomic status (p = 0.018; low/lower middle highest); age was non-significant (p = 0.27). Parental awareness differed by education (p = 0.006; high best) and SES (p=0.009; upper better), not school (p = 0.21). Management scores showed age (p = 0.046; late best), sex (p = 0.030; boys better), and education effects (p = 0.033; higher best). These highlight disparities in stress burden among girls, nuclear families, and lower socioeconomic status adolescents. Post hoc analysis indicated that stress scores differed significantly between primary and higher education groups (p = 0.006), whereas differences between primary and secondary, or secondary and higher, groups were not significant. For parental awareness, all pairwise comparisons were statistically significant, with more-educated parents demonstrating greater awareness. In terms of stress management, a significant difference was observed between primary and higher education groups (p = 0.001), whereas other comparisons were not statistically significant. Overall, higher parental education was associated with lower stress levels and better awareness and coping abilities.

**Table 4:** Comparison of outcome score by key variables. *P<0.05 was considered statistically significant. t = independent t-test was applied for sex, school type, family type, and socioeconomic status, f = one-way analysis of variance (ANOVA) test was applied for age group and parental education. ADOSS: Adolescent Stress Scale; BRS-6: Brief Resilience Scale

Characteristics	Category	Stress score (ADOSS)		Parental awareness score		Stress management score (BRS-6)	
		Test value	p-Value	Test value	p-Value	Test value	p-Value
Age group (years)	Early (11‑12)	f = 1.32	0.27	f = 0.31	0.73	f = 3.12	0.046*
	Middle (13‑16)						
	Late (17‑18)						
Sex	Adolescent boys	t = 2.45	0.015*	t = 0.64	0.52	t = 2.18	0.030*
	Adolescent girls						
School type	Government school	t = 3.74	0.001*	t = 1.63	0.21	t = 1.85	0.31
	Private school						
Family type	Joint family	t = 2.12	0.035*	t = 1.12	0.26	t = 1.23	0.22
	Nuclear family						
Parental education	Primary	f = 3.67	0.027*	f = 5.23	0.006*	f = 3.45	0.033*
	Secondary						
	Higher						
Socioeconomic status	Low/lower middle	t = 4.12	0.018*	t = 4.89	0.009*	t = 1.76	0.13
	Upper middle/high						

Multivariate regression for stress score explained 32% of the variance in stress scores (p<0.001). Lower parental awareness strongly predicted higher stress (p = 0.001), followed by female gender (p = 0.015), nuclear family (p = 0.035), private school (p = 0.021), lower socioeconomic status (p = 0.015), and low parental education (p = 0.020). Higher awareness, socioeconomic status, and education buffered stress, while female sex and nuclear family structure increased stress compared with the reference groups (male sex, government employment, joint family structure, and low socioeconomic status). These predictors underscore modifiable factors like awareness of interventions, critical in society to curb stress-linked disorders in vulnerable adolescents (Table 5).

**Table 5:** Multivariate regression analysis for predictors of stress score in adolescents. *P<0.05 was considered statistically significant. The multivariate regression model explained 32% of the variance in adolescent stress scores, and this model was statistically significant (R² = 0.32, p<0.001). CI: confidence interval at 95% (lower value, upper value); SES: socioeconomic status

Predictor	β coefficient	Standard error	β 95% CI	t-Value	p-Value
Parental awareness	-0.35	0.08	-0.50, -0.20	-5.62	0.001*
Sex (female)	0.18	0.09	0.01, 0.35	2.45	0.015*
School type (private)	0.14	0.07	0.01, 0.27	2.62	0.021*
Family type (nuclear)	0.15	0.07	0.01, 0.29	2.12	0.035*
Parental education (higher)	-0.12	0.06	-0.23, -0.01	-2.34	0.020*
SES (higher)	-0.14	0.07	-0.27, -0.01	-2.45	0.015*

Multivariate regression for stress management accounted for 38% of the variance in stress management scores (p<0.001). Higher parental awareness (p = 0.001) and lower stress scores (p = 0.001) were the strongest positive predictors. High parental education (p = 0.015), late adolescence (p = 0.029), and male sex buffered deficits versus references. Female sex negatively impacted coping (p = 0.030). These bidirectional stress-management links emphasize awareness training to enhance resilience, particularly for girls and early adolescents (Table 6).

**Table 6:** Multivariate regression analysis for predictors of stress management score in adolescents. *P<0.05 was considered statistically significant. The multivariate regression model explained 38% of the variance in adolescent stress scores, and this model was statistically significant (R² = 0.38, p<0.001). CI: confidence interval at 95% (lower value, upper value).

Predictor	β coefficient	Standard error	95% CI	t-Value	p-Value
Parental awareness	0.32	0.07	0.19, 0.45	5.12	0.001*
Stress score	-0.28	0.06	-0.39, -0.17	-4.67	0.001*
Sex (female)	-0.16	0.08	-0.31, -0.01	-2.18	0.030*
Parental education (higher)	0.13	0.06	0.02, 0.24	2.45	0.015*
Age group (late)	0.11	0.05	0.01, 0.21	2.20	0.029*

## Discussion

The present study aimed to investigate adolescent stress, its management, and the influence of parental awareness on these outcomes. The findings of this study provide important insights into the complex interplay between individual, familial, and socioenvironmental factors influencing adolescent mental health. Adolescent stress is a multifactorial phenomenon influenced by academic, social, and psychological pressures. The significant association observed between stress levels and demographic variables such as gender, family type, parental education, and socioeconomic status highlights the multidimensional nature of stress. This finding aligns with the work of Wu, who emphasized that adolescent mental health is shaped by multiple stressors, including family environment, academic expectations, and social dynamics [15]. Similarly, Anjana reported that stress among youth is strongly influenced by external environmental factors, particularly familial and socioeconomic conditions [16]. These studies support the present findings, suggesting that stress is not an isolated construct but is deeply embedded in contextual realities.

The observed sex differences in stress levels, although modest, are consistent with previous literature indicating that female adolescents tend to report higher stress due to greater emotional sensitivity and social expectations. Studies such as those by Khadka et al. have demonstrated that parenting styles and emotional climate within the family significantly influence psychological outcomes such as stress and anxiety, particularly among female adolescents [10]. This supports the notion that sex-specific interventions may be necessary for effective stress management.

Parental education and socioeconomic status were found to be significantly associated with both stress and parental awareness. This is consistent with the findings of Li's study, which highlighted that educated parents are more likely to adopt supportive parenting practices, thereby positively influencing adolescent mental health [17]. Higher socioeconomic status often provides better access to resources, emotional support systems, and educational opportunities, which collectively reduce stress levels. These findings are further supported by Yang et al., who demonstrated that family support and social resources play a critical role in enhancing mental well-being among adolescents [8].

A key finding of the present study is a significant association between parental awareness and stress management among adolescents. This underscores the critical role of parents in shaping coping mechanisms. Putri and Dariyo found a strong correlation between parental involvement and effective stress coping strategies among students, reinforcing the present findings [18]. Similarly, Zimmer-Gembeck et al. reported that parental support significantly enhances adolescents’ ability to cope with academic stressors, independent of academic pressure and achievement levels [9]. These findings suggest that parental awareness acts as a protective factor, enabling adolescents to develop adaptive coping strategies.

The relationship between stress management and demographic variables such as age, sex, and parental education further highlights developmental and environmental influences. Older adolescents demonstrated better stress management, which may be attributed to increased maturity and experience in handling stressors. This is consistent with the findings of Jeyasingh, who reported that coping strategies evolve with age and experience [7]. Additionally, structured stress management interventions, as demonstrated by Kale and Shula, have been shown to significantly improve adolescents’ coping abilities, suggesting that both intrinsic and extrinsic factors contribute to stress management [19].

The strong relationship observed between parental awareness and stress management further validates the theoretical framework that emphasizes the family as a primary socializing agent. Ukhanova demonstrated that parental involvement moderates the relationship between stressful events and adolescent mental health, indicating that awareness and engagement can buffer the negative effects of stress [20]. Similarly, Ramlan and Ahmad reported that parental involvement is positively associated with academic resilience and inversely related to stress levels, reinforcing the mediating role of family dynamics [21].

Moreover, the findings of this study align with community-based mental health initiatives such as those described by Puspitasari and Rohmah, which emphasize the importance of family and community support in improving adolescent mental health outcomes [1]. Chronic stress during adolescence has been shown to adversely affect academic performance and long-term psychological well-being, as reported by Schraml et al., further highlighting the need for early identification and intervention [5].

Despite the strengths of this study, certain limitations must be acknowledged. The cross-sectional design limits the ability to establish causal relationships between variables. Self-reported measures may introduce response bias, particularly in sensitive areas such as stress perception and parental awareness. Additionally, the study was conducted within a specific geographical region, which may limit the generalizability of the findings to other populations. The use of resilience as a proxy measure for stress management, although methodologically justified, may not fully capture the multidimensional nature of coping strategies. Furthermore, unequal subgroup sizes within certain demographic categories may reduce statistical power.

Future research should consider longitudinal designs to better understand causal pathways and temporal relationships between parental awareness, stress, and coping mechanisms. Inclusion of qualitative components could provide deeper insights into adolescents’ lived experiences. Expanding the study across diverse cultural and socioeconomic settings would enhance external validity.

## Conclusions

The present study reinforces the critical role of parental awareness in shaping adolescent stress and stress management. It highlights the need for family-centered interventions and policy-level strategies aimed at improving parental engagement and awareness. Addressing adolescent stress requires a holistic approach that integrates individual, familial, and societal dimensions, thereby promoting overall mental well-being.

## Appendices

**Table 7:** Parental awareness questionnaire. Scoring system - minimum score: 25, maximum score: 75. Interpretation - low awareness: 25-34, moderate awareness: 35-63, high awareness: 64-75.

Title: Effect of parental awareness on adolescent stress management	
Instructions: please indicate how often you follow each of the statements below in relation to your adolescent child	
Response options: never (1), sometimes (2), always (3)	Response
Section A: awareness of adolescent stress (items 1-8)	
1. I am aware that adolescents experience academic stress.	
2. I understand that peer pressure can affect my child’s mental health.	
3. I recognize signs of stress such as irritability or withdrawal.	
4. I am aware that excessive screen time can increase stress.	
5. I know that sleep disturbances may indicate stress in my child.	
6. I am aware that emotional changes during adolescence are normal.	
7. I recognize that exam periods can increase anxiety levels.	
8. I am aware of the impact of social media on my child’s stress.	
Section B: communication and emotional support (items 9-16)	
9. I encourage my child to talk about their feelings.	
10. I listen actively when my child shares concerns.	
11. I avoid criticizing my child when they are stressed.	
12. I provide reassurance during difficult situations.	
13. I maintain open communication with my child.	
14. I spend quality time with my child regularly.	
15. I support my child in managing emotional challenges.	
16. I create a safe environment for my child to express themselves.	
Section C: awareness of stress management strategies (items 17-21)	
17. I encourage my child to engage in physical activities.	
18. I promote healthy habits such as proper sleep and diet.	
19. I am aware of relaxation techniques (e.g., meditation, breathing).	
20. I guide my child to balance academics and leisure activities.	
21. I encourage hobbies to reduce stress.	
Section D: monitoring and professional support (items 22-25)	
22. I monitor changes in my child’s behavior regularly.	
23. I communicate with teachers regarding my child’s well-being.	
24. I seek professional help when needed (e.g., counselor).	
25. I am aware of mental health resources available for adolescents.	
